# Nurses’ use of social media during the COVID-19 pandemic—A scoping review

**DOI:** 10.1371/journal.pone.0263502

**Published:** 2022-02-18

**Authors:** Stinne Glasdam, Helena Sandberg, Sigrid Stjernswärd, Frode F. Jacobsen, Anette H. Grønning, Lisbeth Hybholt

**Affiliations:** 1 Department of Health Sciences, Faculty of Medicine, Lund University, Lund, Sweden; 2 Media and Communication Studies, School of Health and Welfare, Halmstad University, Halmstad, Sweden; 3 Department of Communication and Media, Faculty of Social Sciences, Lund University, Lund, Sweden; 4 Centre for Care Research Western Norway, Western Norway University of Applied Services Bergen, Bergen, Norway; 5 VID Specialized University, Stavanger, Norway; 6 Media Studies, Department for the Study of Culture, University of Southern Denmark, Odense, Denmark; 7 Centre for Relationships and De-escalation, Mental Health Services Region Zealand, Slagelse, Denmark; University of Haifa, ISRAEL

## Abstract

**Background:**

During the COVID-19 pandemic, nurses stand in an unknown situation while facing continuous news feeds. Social media is a ubiquitous tool to gain and share reliable knowledge and experiences regarding COVID-19. The article aims to explore how nurses use social media in relation to the COVID-19 pandemic.

**Method:**

A scoping review inspired by Arksey and O’Mally was conducted by searches in Medline, CINAHL, Academic Search Complete and Web of Sciences. Empirical research studies investigating nurses’ use of social media in relation to COVID-19 were included. Exclusion criteria were: Literature reviews, articles in languages other than English, articles about E-health, and articles investigating healthcare professionals without specification of nurses included. Articles, published in January-November 2020, were included and analysed through a thematic analysis. The PRISMA-ScR checklist was used.

**Results:**

Most of the eleven included studies were cross-sectional surveys, conducted in developing countries, and had neither social media nor nurses as their main focus of interest. Three themes were identified: ‘Social media as a knowledge node’, ‘Social media functioned as profession-promoting channels’ and ‘Social media as a disciplinary tool’. Nurses used social media as channels to gain and share information about COVID-19, and to support each other by highlighting the need for training and changes in delivery of care and redeployment. Further, social media functioned as profession-promoting channels partly sharing heroic self-representations and acknowledgment of frontline persons in the pandemic, partly by displaying critical working conditions. Finally, nurses used social media to educate people to perform the ‘right ‘COVID-19’ behaviours in society.

**Conclusion:**

This review provided snapshots of nurses’ uses of social media from various regions in the world, but revealed a need for studies from further countries and continents. The study calls for further multi-methodological and in depth qualitative research, including theoretically framed studies, with a specific focus on the uses of social media among nurses during the pandemic.

## Introduction

There is a global shortage of healthcare professionals, in particular nurses [1, 2]. The World Health Organization (WHO) [2] estimates that the world will need an additional 9 million nurses and midwives by 2030, and Scheffler and Arnold [3] project a shortage of nearly 2.5 million nurses across 23 OECD countries in 2030. Several studies show that perceived staffing adequacy, both in numbers and qualifications, is of major importance for nurses to leave or stay at a workplace [4, 5]. Today, nurses’ work situation is affected by the current coronavirus SARS-CoV-2 and COVID-19 outbreak in multiple ways, with an increased workload, safety issues, novel and constantly updated work routines, an unpredictable future, feelings of worry and insecurity, and more [6–8].

Nurses’ feelings of worry and insecurity can be linked to the overwhelming crisis situation caused by the COVID-9 pandemic [9], as it is important for healthcare professionals to be well prepared to take care of patients with COVID-19 [10, 11]. Care provision is a considerable mental and emotional challenge during a pandemic, which when combined with exhaustion may result in nurses being infected, dying, getting burned-out and/or leaving their jobs [12–15]. Nurses’ commitment to caring for their patients is hence often detrimental to their own mental and physical wellbeing [8, 13, 16]. The negative impact of COVID-19 outbreaks on health professionals’ mental health is confirmed by further studies, e.g. through the nurses’ repeated and prolonged exposure to stressors due to direct exposure to infectious diseases [11, 17–19]. Also, insomnia and sleep disorders are shown among healthcare professionals during the pandemic [8].

During the current pandemic, nurses stand on precarious ground, in an unknown situation and with continuous news feeds [6]. It is important and challenging for nurses to be up to date with reliable knowledge and share experiences with each other to handle this new situation. Social media has become a ubiquitous tool for an increasing number of people in multiple areas of daily life. Social media enables private and professional uses, functioning as a source of e.g. news and information, entertainment, opinion making, networking, and connectivity, as well as identity construction [20–23], also for healthcare professionals with clinical positions [24–26]. Social media can be defined as internet-based applications for user-generated content production, sharing and communication where individuals and groups create user-specific profiles for a site or app designed and maintained by a social media service [27, 28]. Social media is an umbrella term covering technologies, services, platforms and channels. Social media technologies allow users to co-create, distribute and share information at different levels of participation, facilitating ‘prosumption’, understood as the blurring of production and consumption, of information in contrast to only consuming e.g. COVID-19 information online [29–31]. Social media services further facilitate the development of social networks online by connecting a profile with those of other individuals and/or groups [32]. Examples of social media platforms include Twitter, Facebook, Instagram, LinkedIn, YouTube, and Clubhouse, etc. Social media channels can be sources of all kinds of information [33, 34]. They offer moments of entertainment, encouragement, and relaxation during the day. It is unknown how, for what purposes, and to what extent nurses use social media for knowledge search, knowledge production and knowledge sharing, as well as for the exchange of experiences and emotions related to the direct work with patients during this pandemic. A crisis like the current one with COVID-19 strongly affects the nurses’ work situation, including the physical, mental and social work environment. There are enhanced stress and suffering in patients and their families, challenging safety and ethical issues, novel and constantly updated work routines, and an unpredictable future with subsequent feelings of worry and insecurity, to name a few aspects [6, 7]. This may lead to serious consequences on sustainability of nurses’ work life. An understanding of whether and how nurses relate to the vast amount of information pervading the multitude of popular social media platforms may help us understand if and how nurses navigate and make use of social media in their professional role and whether such use can be an empowering or disempowering factor. News travels fast and on a global scale through social media [35], contributing to a rapid spread of all kinds of information to wide audiences. As social media can be a source of both emotional turmoil [21] and emotional support [36], it is of particular importance to explore its potential uses in the current COVID-19 pandemic. Therefore, this scoping review aims to explore how nurses use social media in relation to the COVID-19 pandemic.

## Method

A scoping review inspired by Arksey and O’Mally [37] was conducted in November 2020—January 2021. Scoping reviews are useful for examining emerging evidence. This approach seemed relevant as the literature in this field was characterised by studies with a wide range of designs and the research question became relatively broad [37]. The method of this scoping review will be presented below in five stages, inspired by Arksey and O’Mally [37]: 1) Identifying the research question, 2) identifying relevant studies, 3) study selection, 4) charting the data and 5) analytical strategy. The Prisma-ScR checklist was used for reporting this review [38], see S1 Checklist. The review was not registered and a protocol was not prepared.

### Identifying the research question

Overall, we identified two research questions: 1) What is the scope of the empirical research on how nurses use social media in relation to the COVID-19 pandemic? and 2) How are the nurses’ uses of social media described?

### Identifying relevant studies

The two broad research questions aim at examining the extent, range and nature of relevant research activity and to identify types of available evidence of relevance to the topic of this article. The method of scoping review is considered an apt method for achieving these aims. A systematic literature search was conducted in four databases: CINAHL, Medline, Academic Search Complete (EBSCO) and the Web of Science citation database. We chose these databases as they provide the opportunity to find articles that have been published in both the health and media fields. In each of the three selected databases, the search strategy consisted of a building block search carried out according to the population, exposure and outcome (PEO) model [39]; 1) Population: nurses, 2) Exposure: Social media, and 3) Outcome/Theme: COVID-19, see (Table 1).

**Table 1:** Populations, exposures and outcomes, PEO.

Block 1 (P)–Population	Block 2 (E)—Exposure	Block 3 (O)—Outcome/Theme
Nurse* OR Health care professional* OR Health care worker* OR Healthcare professional* OR Healthcare worker*	Social media OR Facebook OR Messenger OR TikTok OR WeChat OR Instagram OR QZone OR Weibo OR Twitter OR LinkedIn OR YouTube OR WhatsApp OR Snapchat OR Pinterest OR Viber OR Reddit OR Discord	COVID-19 OR Corona virus

Each block included a variation of relevant search terms. The search was limited to articles written in English and published from 1st January 2020 - 7th November 2020, closely related to the onset of the COVID-19 pandemic and beyond. Table 2 shows the full electronic search strategy used to identify studies with all search terms and limits for all three databases.

**Table 2:** The full electronic search strategy for all three databases.

Database	Permalink resp. search string
CINAHL	http://search.ebscohost.com.ez-sus.statsbiblioteket.dk:2048/login.aspx?direct=true&db=c8h&bquery=(+TX+COVID-19+OR+TX+Corona+virus+)+AND+(+TX+Nurse*+OR+TX+Health+care+professional*+OR+TX+Health+care+worker*+OR+TX+Healthcare+professional*+OR+TX+Healthcare+worker*+OR+TX+Health+professional*+OR+Health+worker+*+OR+(MM+%26quot%3bNurses%2b%26quot%3b)+)+AND+(+TX+(+Facebook+OR+Messenger+OR+TikTok+OR+WeChat+OR+Instagram+OR+QZone+OR+Weibo+OR+Twitter+OR+LinkedIn+OR+YouTube+OR+WhatsApp+OR+Snapchat+OR+Pinterest+Or+Viber+OR+Reddit+Or+Discord+)+OR+TX+(+(+(MH+%26quot%3bSocial+Media%2b%26quot%3b)+OR+%26quot%3bSocial+media%26quot%3b+)+OR+TX+Social+media+)+)&cli0=DT1&clv0=201901-202012&type=1&searchMode=Standard&site=ehost-live
Academic Research Complete	https://web-a-ebscohost-com.ludwig.lub.lu.se/ehost/searchhistory/PrintSearchHistory?vid=5&sid=8094cf45-1a56-4955-befb-b96b5879c95d%40sdc-v-sessmgr03&bquery=covid-19+OR+corona+virus&bdata=JkF1dGhUeXBlPWlwLHVpZCZkYj1hOWgmY2xpMD1EVDEmY2x2MD0yMDE5MDEtMjAyMDEyJmNsaTE9UFoxJmNsdjE9QXJ0aWNsZSZjbGkyPUxBOTkmY2x2Mj1FbmcmdHlwZT0xJnNlYXJjaE1vZGU9QW5kJnNpdGU9ZWhvc3QtbGl2ZQ%3d%3d&theSearchHistoryIds
PubMed	("facebook"[All Fields] OR ("messenger"[All Fields] OR "messengers"[All Fields]) OR "TikTok"[All Fields] OR "WeChat"[All Fields] OR "Instagram"[All Fields] OR "QZone"[All Fields] OR "Weibo"[All Fields] OR ("twitter"[All Fields] OR "twitter s"[All Fields] OR "twitters"[All Fields]) OR "LinkedIn"[All Fields] OR ("youtube"[All Fields] OR "youtube s"[All Fields]) OR "WhatsApp"[All Fields] OR "Snapchat"[All Fields] OR ("Pinterest"[All Fields] AND "Or"[All Fields] AND "Viber"[All Fields]) OR ("Reddit"[All Fields] AND "Or"[All Fields] AND ("discord"[All Fields] OR "discords"[All Fields])) OR ("social media"[MeSH Terms] OR ("social"[All Fields] AND "media"[All Fields]) OR "social media"[All Fields]) OR ("social media"[MeSH Terms] OR ("social"[All Fields] AND "media"[All Fields]) OR "social media"[All Fields])) AND ("severe acute respiratory syndrome coronavirus 2"[Supplementary Concept] OR "severe acute respiratory syndrome coronavirus 2"[All Fields] OR "ncov"[All Fields] OR "2019 ncov"[All Fields] OR "covid 19"[All Fields] OR "sars cov 2"[All Fields] OR (("coronavirus"[All Fields] OR "cov"[All Fields]) AND 2019/11/01:3000/12/31[Date—Publication]) OR (("corona"[All Fields] OR "coronae"[All Fields] OR "coronas"[All Fields]) AND ("virology"[MeSH Subheading] OR "virology"[All Fields] OR "viruses"[All Fields] OR "viruses"[MeSH Terms] OR "virus s"[All Fields] OR "viruse"[All Fields] OR "virus"[All Fields]))) AND ("nurses"[MeSH Terms] OR ("health personnel"[MeSH Terms] OR ("health"[All Fields] AND "personnel"[All Fields]) OR "health personnel"[All Fields] OR ("health"[All Fields] AND "care"[All Fields] AND "professional"[All Fields]) OR "health care professional"[All Fields]) OR ("health personnel"[MeSH Terms] OR ("health"[All Fields] AND "personnel"[All Fields]) OR "health personnel"[All Fields] OR ("health"[All Fields] AND "care"[All Fields] AND "worker"[All Fields]) OR "health care worker"[All Fields]) OR (("delivery of health care"[MeSH Terms] OR ("delivery"[All Fields] AND "health"[All Fields] AND "care"[All Fields]) OR "delivery of health care"[All Fields] OR "healthcare"[All Fields] OR "healthcare s"[All Fields] OR "healthcares"[All Fields]) AND "professional*"[All Fields]) OR ("TX"[All Fields] AND ("health personnel"[MeSH Terms] OR ("health"[All Fields] AND "personnel"[All Fields]) OR "health personnel"[All Fields] OR ("healthcare"[All Fields] AND "worker"[All Fields]) OR "healthcare worker"[All Fields])) OR ("TX"[All Fields] AND ("health personnel"[MeSH Terms] OR ("health"[All Fields] AND "personnel"[All Fields]) OR "health personnel"[All Fields] OR ("health"[All Fields] AND "professional"[All Fields]) OR "health professional"[All Fields])) OR (("health"[MeSH Terms] OR "health"[All Fields] OR "health s"[All Fields] OR "healthful"[All Fields] OR "healthfulness"[All Fields] OR "healths"[All Fields]) AND ("occupational groups"[MeSH Terms] OR ("occupational"[All Fields] AND "groups"[All Fields]) OR "occupational groups"[All Fields] OR "worker"[All Fields] OR "workers"[All Fields] OR "worker s"[All Fields])))

The 738 identified studies were transferred to COVIDENCE.org for the following screening process. We supplemented the building block with a citation pearl search in the Web of Science citation database of the included articles to be able to find relevant studies outside the three databases. We did not include grey literature defined as all kinds of materials produced by governments, academics, business and industry, which is not controlled by commercial publishers [40] as the focus was on peer reviewed research articles within the topic.

### Study selection

**Fig 1:**
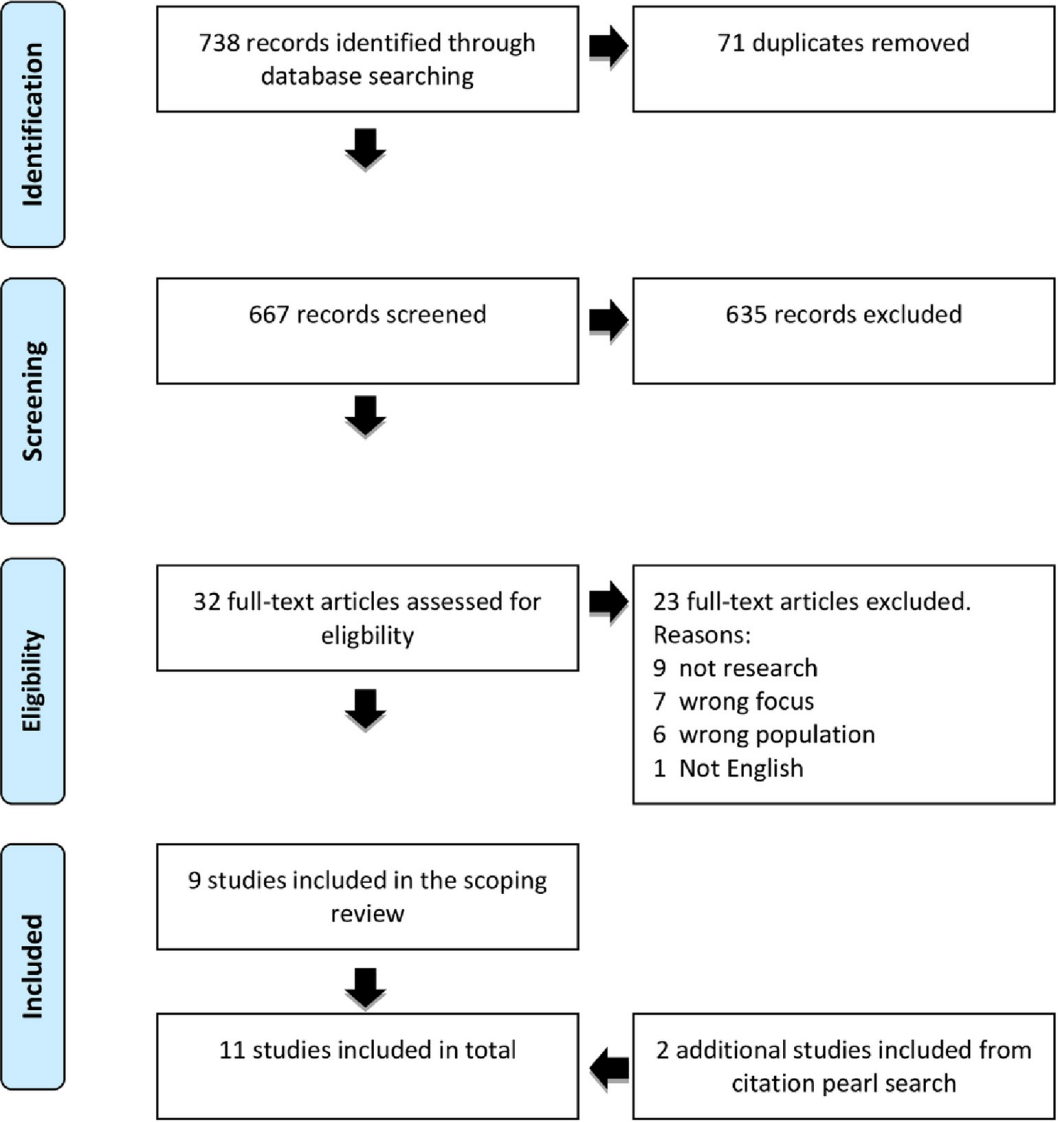
PRISMA flow diagram.

The first and last authors (SG & LH) of the current article separately screened the article titles, abstracts and full texts using COVIDENCE.org with the following criteria: empirical research studies investigating nurses’ use of social media in relation to COVID-19. Inclusion criteria: All articles investigating or presenting results on how nurses used social media during the COVID-19 pandemic were included, as for example investigations on COVID-19 knowledge among nurses and social media as a source of knowledge. The following articles were excluded: Literature reviews, articles in languages other than English, articles about E-health, and articles investigating healthcare professionals without specification of nurses included. In case of disagreement in the screening process, the two authors discussed the inclusion/exclusion until agreement was reached. The review included nine articles. Further, two articles were included after the citation pearl search in Web of Science. The study selection process is summarised in the PRISMA flow chart [41], see Fig 1.

### Charting the data

The third author (SS) completed the review’s data extraction and coding stages. A structured data extraction spreadsheet was created to extract data from the included studies based on the Cochrane Consumers and Communication Review Groups data extraction template [42]. The following information was extracted from the articles: 1) Authors, 2) Country, 3) Journal, 4) Impact factor (extracted from journal website), 5) Study period, 6) Study design, 7) Sample size, 8) Target group and context, 9) Theory, 10) Results, and 11) Limitations. A selection of the data is presented in Table 2. The theories, results and limitations are presented and discussed in the body of the text in the Results section and in the thematic analysis that follows. The extracted data was checked for accuracy and further discussed by two of the authors (HS & SS). The validation of the extracted data could be described as an iterative process in which comparisons were made repeatedly between the extracted data and the original articles. In case there were discrepancies or important nuances missing in the table, the data was further discussed and, if necessary, complemented until a consensus was reached among the authors. In line with Arksey and O’Mally [37], the current scoping review does not include a quality assessment of the studies. Two of the authors (HS & SS) nonetheless charted the studies’ strengths and limitations as reported by the respective articles’ authors. This process was first done independently, then jointly, and potential discrepancies were again resolved through in depth discussions until a mutual agreement was reached.

### Analytical strategy

The analysis was a collating and summarising of the relevant results. As in scoping reviews in general, the analysis aimed to create an overview or map of the types of evidence and knowledge in a given field [43, 44]. Levac and colleagues [45] recommended three distinct steps in the analytical phase of a scoping review: 1) analysing the data including a descriptive numerical summary analysis and a qualitative thematic analysis, 2) reporting the outcome articulated through themes referring to research questions of the study, and 3) applying meaning to the results relating to the overall aim of the study. In this study, the descriptive summary analysis is the results of the data charting, and Evans [46] inspired the qualitative thematic analysis. The descriptive summary analysis was presented as ‘Characteristics of the studies’ in the result section. Regarding the thematic analysis, first, the articles were read and re-read to develop a sense of the studies as a whole. Second, differences between the studies’ key findings related to the research questions and the aim of the study were compared and contrasted, and three themes were identified by grouping and categorising the findings into areas of similarity. Third, the themes were re-examined to interpret the content of each theme, and to identify consistencies and incongruities. Fourth, the themes described, consisting of ‘Social media as knowledge nodes’, ‘Social media as profession-promotion’ and ‘Social media as a disciplinary tool’.

## Results

This section will start with the characteristics of the included studies in the form of a descriptive summary analysis. Next, the themes will be presented.

### Characteristics of the studies

A schematic overview of the included studies is presented in Table 3.

**Table 3:** Overview of the included studies.

Authors	Country	Journal	Impact factor	Study design	Sample size N = total population asked n = responses	Target group and context
Asemahagn (2020) [47]	Ethiopia	Tropical Medicine and Health	Cite score 2019: 2,5 (Scopus)	Cross-sectional online survey	N = 442 n = 398	Healthcare professionals in public hospitals and health care centres
El-Awaisi et al. (2020) [48]	Qatar (study context not specified, however limited to transnational anglosaxon community on social media)	Journal of Interprofessional Care	(2019:1.726)	Cross-sectional review of social media comments	N = 40 n = 21	Social media posts (21) (across LinkedIn, Twitter, Facebook) including a limited number of associated comments (1576 out of 1759)
Elhadi et al. (2020) [49]	Libya	Am. J. Trop. Med. Hyg.	(2019: 2.126)	Cross-sectional paper-based survey	N = 2000n = 1572	Healthcare professionals from 21 different hospitals in Libya
Forte & Pires (2020) [50]	Brazil	Revista Brasileira da Enfermagem (Supplementary Edition 2)	(2017–2018:0.57)	A qualitative, descriptive, and exploratory study of social media posts.	N = 295 postings n = 295	Social media postings on Twitter and Instagram
Hassan et al. (2020) [51]	Pakistan	Annals of King Edward Medical University (short communication)	NA	Cross-sectional online survey	N = 384 (planned sample size) n = 257	Healthcare professionals in 5 tertiary healthcare facilities in Peshawar, Khyber Pakhtunkhwa
Huynh et al. (2020) [52]	Vietnam	Asian Pacific Journal of Tropical Medicine	(2019: 1.94)	Cross-sectional survey (not specified if online or paper-based)	N = 375 n = 327	Healthcare professionals at District 2 Hospital in Ho Chi Minh City, Vietnam
Paul et al. (2020) [53]	India	Journal of Anaesthesiology Clinical Pharmacology	(2018: 1.25)	Cross-sectional online survey	n = 1026 out of which 558 HCW and 468 GPP	Healthcare professionals (including doctors, nurses, and para-medical staff) and general public participants (GPPs) from a database of medical professionals and personal social networks.
Qadah (2020) [54]	Saudi Arabia	J Infect Dev Ctries	(2019–2020: 1.260)	Cross-sectional online survey	n = 1023	Healthcare professionals (physicians, nurses, pharmacists, technical staff, administrative staff in clinical settings)
Salman et al. (2020) [55]	Pakistan	J Infect Dev Ctries	(2019–2020: 1.260)	Cross-sectional survey (most likely paper-based)	N = 458 n = 429 (convenience sampling method)	Health professionals (medical doctors, nurses, pharmacists, physiotherapists, hospital technicians and technologists) from 7 hospitals in Pakistan.
Sharma et al. (2020) [56]	India	Journal of Clinical and Diagnostic Research	(2019–2020: 0.810)	Cross-sectional paper-based survey	N = 180 n = 164	Health care professionals including doctors (43.9%) and nurses (56.1%) from the medical college hospital, i.e. the Dept of Respiratory Medicine, Jaipur National University Institute for Medical Sciences and Research Centre, Jaipur, India.
Vindrola-Padros et al. (2020) [57]	United Kingdom	BMJ Open	(2019: 2.496)	Multimethodological, qualitative approach	1.Policy review (1 Dec 2019–20 April 2020) (n = 35 UK healthcare policies) 2.Media analysis (1 Dec 2019–30 Apr 2020) (n = 101 newspapers articles + n = 146000 social media posts rom Reddit, Facebook, Instagram and Youtube) 3.Frontline staff interviews (n = 30)(Apr 2020)	Policies (selection from legislation.gov.uk, gov.uk, NHSE and PHE databases), Media (newspaper articles, social media posts), Frontline staff in emergency departments and intensive care units in 3 hospitals in London (nurses, doctors, allied health professionals)

All the studies were carried out in the first six months of 2020, in a diversity of countries representing multiple regions such as Africa, Asia, the Middle East, South America, and Europe. The studies took place in Brazil, Ethiopia, India (2), Libya, Pakistan (2), Qatar, Saudi Arabia, the United Kingdom, and Vietnam. Most studies were published in journals with a focus on developing countries, infectious diseases, tropical medicine, clinical care, or health professions. The journals’ impact factor (IF) ranged from 0.5–2.5 (some with no impact reported).

The studies’ research areas and aims consisted of evaluations of risk perception, attitudes, knowledge and preparedness related to COVID-19 [47, 49, 51–56] and explorations of social media posts [48, 50], including policies, and both social media and traditional printed news media and magazines [57].

Nine out of eleven studies were descriptive cross-sectional surveys (n = 9; seven online, one paper-based, one unclear). A majority targeted healthcare professionals (n = 9) and/or media content such as social media posts (n = 3) [48, 50, 57], and policy documents and news coverage (n = 1) [57]. Data was collected through the means of validated and non-validated questionnaires, the sampling of (social) media posts and policies, and interviews.

The population studied, i.e. the samples and platforms (e.g. social media posts) in the studies consisted of healthcare professionals including nurses, physicians, paramedical staff, pharmacists, physiotherapists, and other clinical and administrative staff from a variety of healthcare related contexts [47, 49, 51–56], members of the general public [53], social media posts from platforms such as Twitter, YouTube, Instagram, LinkedIn, Reddit and Facebook [48, 50, 57], and policies and media (social media, traditional media) [57].

Social media did not come through as the major object of study in most studies. Only one article used social media in the title [48]. Only one article had social media as one out of several keywords [48] and another had ‘communications media’ as a keyword [50]. All articles mention social media in their abstracts, either in terms of a distribution channel, social media as data collection method or as an object of study (a variable), e.g. general uses of social media, social media as a source of information, user generated content on social media, or nurses’ uses of social media to express themselves. Forte and Pires’ [50] study illustrated a clear bottom-up perspective with social media as a medium for self-expression and interaction, in comparison to most of the other studies, which had a top-down perspective on communication where social media was treated as a tool for transmission or distribution of information. Three of the articles [48, 50, 57] expressed a more nuanced understanding of social media. All but the three former articles focused on determining, assessing, or evaluating knowledge, attitudes, perceptions, and/or preventive practices and preparedness where nurses’ use of social media appeared as a sub-element (one out of many explanatory or independent variables to explain attitudes or behaviour) in the study, exemplified by titles such as: “*Assessment of knowledge*, *attitude and practice regarding COVID-19…”* [56] and *“Factors determining the knowledge and prevention practice of healthcare workers towards COVID-19…*.*”* [47].

Most studies did not refer to any theories. Three of the articles referred to theoretical frameworks [47, 50, 57], but the theories did not reappear or drive the result analysis nor the discussion. The use of the theories was thus shrouded in obscurity. Key concepts such as knowledge, attitudes, risk, perceptions, media use, preparedness etc., were often taken for granted and consequently not properly defined or discussed in detail. The descriptions of the survey instruments (the questionnaires) were in some cases poor (e.g. [49, 55]) lacking in detail and transparency, with scarce information of items (variables) and questions asked, making it difficult to not only interpret the results but also to assess their significance. Two studies included the full questionnaire as appendix [53, 56], and one referred to supplementary material to be retrieved through an URL-link [47]. There was generally a lack of reflection about the study’s methods and brief comments on the limitations. One study does not report any limitations [54]. All other studies report a varied number of limitations. The cross-sectional studies for instance reported limitations pertaining to the samples’ characteristics and size and thus representativity, to the chosen distribution method, which may prevent e.g. people without Internet access from participating, to single/limited study contexts, and e.g. contexts with limited number of known/detected COVID-19 infection, and to risk of bias pertaining to (lack of) social media access. Further, no causal inferences can be made due to the studies’ design. Further reported limitations were associated with the studies’ time frame and with weaknesses in the design of the measurement instruments and operationalisation of the study objects, such as limited numbers of survey items related to the measured concepts. The non-cross-sectional studies [48, 48, 57] also reported a number of limitations. El Awaisi [48] for instance reported a limited knowledge of the samples’ sociodemographic characteristics, limited representativity, risk of bias related to study time frame and data collection period, and encoding bias.

### Social media as knowledge nodes

The results revealed a variety in the healthcare professionals’ use of social media in relation to COVID-19 [47, 49, 51, 52, 54–57]. Some studies found that social media was predominant over other sources of information [49, 52, 55, 56]. Huynh and colleagues [52] found that 91.1% of healthcare professionals, including nurses, in Vietnam used social media to inform themselves about COVID-19, compared to the Ministry of Health Website (82.6%), television (79.2%), and friends and relatives (43.4%). Among healthcare professionals, including nurses, Salman and colleagues [55] also found that social media was the major source (65%) of information in Pakistan, followed by television/radio (24%), friends/family/relatives (7.7%), and newspapers (1.9%). In the study by Sharma and colleagues [56], the most common source of information about COVID-19 among healthcare professionals, including nurses, was the internet (79.3%) followed by social media (69.5%), television (61%) newspapers (59.8%), government sources (58.5%), friends and family (32.9%), and radio (17.1%). According to the studies by Elhadi and colleagues [49] and Sharma and colleagues [56], 65,1% and 70% of healthcare professionals, including nurses, in Libya and India, respectively, used social media as a source of information. The study of Qadah et al. [54] found that 39.8% of the healthcare professionals in Saudi Arabia had heard about COVID-19 through social media.

Overall, social media functioned as channels where healthcare professionals, including nurses, gained information about COVID-19 [47, 49–52, 54–57]. However, in the study of Paul and colleagues [53] only 8% of the healthcare professionals, including nurses, in India relied on social media for information related to COVID‑19. These findings were in contrast to Asemahagn [47] that found that healthcare professionals in Ethiopia who used social media as information sources were 2.5 times more knowledgeable compared to healthcare professionals who did not access information using social media. Several studies showed that a major part of the healthcare professions had relatively good COVID-19 related knowledge [51, 52, 55]. Nevertheless, Elhadi and colleagues [49] found that the majority of healthcare professionals, including nurses, reported an inadequate level of knowledge on COVID-19.

Vindrola-Padros and colleagues [57] found that social media were used by nurses and other healthcare professionals in the United Kingdom to share inconsistencies in COVID-19 advice and in that way sharpened attention to possible issues in the management of COVID-19. Healthcare professionals used social media to support each other through the need for training and changes in delivery of care and redeployment, e.g. by weekly chats via Twitter around specific hashtags with discussions of new COVID-19 procedures in healthcare, sharing of educational/training guidelines, etc. [57].

### Social media as profession-promotion

Social media became channels for heroic self-representations and acknowledgment of frontline persons, also nurses, in the pandemic [48, 50, 57]. El-Awaisi and colleagues [48] showed that the word ‘heroes’ was commonly used in posts on social media. The posts were used as reminders that many healthcare, non-healthcare professionals and volunteers risked their lives and worked around the clock to ensure the safety of COVID-19 ill patients [48] Forte and Pires [50] showed that nurses in Brazil appreciated being honoured and being called heroes, and Vindrola-Padros and colleagues [57] showed that nurses were proud of their jobs and often called on the need to be adaptable, resilient, and flexible through their heroic posts.

Some posts on social media also pointed to acknowledging and recognising overlooked health professions other than nurses and doctors, such as speech therapists and physician assistants for their efforts during the pandemic [48]. Vindrola-Padros and colleagues [57] found that solidarity between colleagues expressed through social media platforms generated positive emotions. In that sense, social media functioned as channels for fights and consolidations of healthcare professions in the medical profession’s hierarchy.

Studies also showed that social media posts were used to display critical working conditions such as ‘worked around the clock’ [48] and needs of personal protective equipment [50]. Forte and Pires [50] also showed that nurses replied to other people’s publications calling nursing professionals heroes, with appeals referring to a workday with precarious working conditions, absence of a decent salary floor and special retirement plan.

### Social media as a disciplinary tool

Nurses used social media to raise awareness among people to the ‘right ‘COVID-19’ behaviours [50]. Forte and Pires [50] showed how nurses made appeals about staying at home during the quarantine period as stipulated by the state and municipal governments. The nurses also used social media posts to sensitise and educate people to remain in their homes, wash their hands correctly, use 70% alcohol for antisepsis, and apply ‘cough etiquette’ as preventive efforts to minimise the spread of COVID-19 [50].

## Discussion

This discussion focuses on three main findings. First, we discuss how social media serves as knowledge nodes for nurses. Second, we discuss how social media is used for profession-promoting purposes. Third, we discuss how nurses use social media to discipline the general public and foster appropriate COVID-19 hygiene and behaviour. Finally, we discuss the strengths and limitations of the scoping review.

The results show that social media serves as knowledge nodes for nurses, mostly in the form of a short statement that nurses gain information about COVID-19 via social media. One article shows that nurses also used social media to support each other through highlighting the need for training and changes in delivery of care and redeployment [57]. The latter is in line with Cheng et al.’s [58] study on peer support and crisis intervention for interdisciplinary teams with nurses utilising an application on smartphones. While social media used for peer-to-peer communication is mentioned, social media used for direct contact with patients and their families is thematically absent in the current review. As online patient-provider, communication may offer a new option for the delivery of affordable health services in a timely manner [59], this could represent an opportunity for nurses to take into account in the development of their professional practice, not the least in times of crisis such as the current COVID-19 pandemic. During the COVID-19 pandemic, studies show how different medical teams use social media (e.g. WhatsApp and Facebook Messenger) to make timely diagnosis of severe cases for prompt medical attention and to prevent the spread of disease by advocating social distancing and masking [60, 61, 70]. Several included studies in current review found that nurses got valuable knowledge through social media [47, 51, 52, 55], but one study found that only 8% of the healthcare professionals in India relied on social media for information related to COVID‑19 [53]. Studies about ethical concerns and power, when using social media suggest that nurses might find it problematic to follow, post, read and trust content on social media [62, 63]. Kuma et al. [64] show that prior positive experiences with social media, e.g. Facebook groups, might significantly influence the trust in such online groups.

Further, the results revealed that social media were used as channels for professional promotion, where both self-representations and a fight for awareness of nurses’ importance, knowledge, competence, work ethics and working conditions were central issues, both in relation to the COVID-19 pandemic and in general. This seems to differ somehow from literature with focus on physicians’ or pharmacists’ uses of social media. Studies reported that most physicians increased their use of social media during the pandemic, with social media being the most important source of COVID-19 information [65, 66]. In addition, pharmacists used and actively encouraged their colleagues to use social media [67]. One study analysing over 10.000 tweets from physicians, concluded that more than 50% of the tweets related to actions, recommendations and concerns about possible misinformation related to COVID-19. Concerns about the healthcare system and working conditions were an actual issue, however, much further down on the list of important topics from the position of physicians [68]. The most prominent topic for the pharmacists studied appeared to be counteracting misinformation, while the theme of working conditions and the need for and plight of this professional group were not mentioned at all. By contrast, the current review stresses the vital importance for nurses, pointing out that they, during the pandemic, put their health and very lives at risk, work more hours and longer work-shifts than strictly obliged to, and suffer from lack of protective measures and gears. Moreover, nurses present themselves, regardless of the COVID-19 pandemic, as a profession with lack of a decent pay and pension plan. Mohammed et al. [69] show that the hero discourse in social media portray nurses as selfless, sacrificing, and outstanding moral subjects for practicing on the front-line under uncertain conditions. In addition, the hero discourse points to nurses as ‘model citizens’, portraying nurses as compliant, hardworking, and obedient subjects opposite to individuals and groups ignoring the medico-political recommendations regarding COVID-19. Moreover, the hero discourse is a tribute to nurses, reconfiguring nursing from mundane and ordinary to an exciting and impactful work [69]. The present pandemic provided nurses as professionals with a unique opportunity to use social media to demonstrate their indispensability, willingness to devote themselves to work for the common good and to accept sacrifices using, both putting themselves forward as heroes and pointing out that others outside the nursing profession acknowledge them as heroes. Social media platforms hence seem to be a place for nurses to strengthen their own professional self-conception and identity, voice concerns and share experiences of suffering, build strong professional collegial solidarity, and to position themselves in the medical professions hierarchy. Why studies on nurses seem to differ regarding social media and prominent topics is a question that probably has more than one answer. The nurses’ use of social media could reflect real differences in working conditions compared to professions like physicians and pharmacists. The nurses included in the current review’s studies could possibly represent jurisdictions where nurses experience particularly challenging working conditions, and in that way, social media functions as an extended mouthpiece for nurses [33]. Another explanation could be that nurses are more willing to share experiences, also painful, with colleagues and to build collegial solidarity as a way to cope with the situation [70]. The pandemic per se offers nurses personal and professional growth opportunities, also through social media uses [71].

Furthermore, the results showed how social media functioned as a disciplining tool where nurses used social media to raise awareness among other people regarding the right ‘COVID-19- behaviour’. This is in line with other studies showing that social media is used by both professionals and laymen to spread facts for educational purposes and raise awareness about the situation’s gravity [72, 73]. Timotijevic [74] shows that the COVID-19 pandemic illustrates the complex interaction between political judgement, ideological orientations of governments, and medical expert advice forming this medico-political discourse. Nurses are regarded as medical experts in relation to COVID-19, which makes their voice particularly legitimate for people who support the medical discourse on COVID-19 and the need for the ‘new normal’ of behaviours [33]. However, what is understood as (true) information, misinformation or disinformation is dependent on the viewpoint of the information consumers and influences potentially affecting their perspectives [33]. In a crisis such as the COVID-19 pandemic, people are more likely to follow an official announcement, or order, than in so-called ordinary social situations [75], where the nurses’ voices in social media are a prolonged voice of the official guidelines regarding COVID-19. Healthcare professionals’, including nurses, use of social media can contribute to rapid dissemination by providing people with the latest medico-political knowledge and useful practices for handling COVID-19 [76]. In that way, social media functions as a channel to both raise people in the right COVID-19 spirit and consolidate the medico-political understanding of (true) information and (true) knowledge [33].

Scoping reviews are especially relevant for mapping research areas such as nurses’ use of social media, with emerging evidence and a lack of randomised controlled trials, as studies with a range of study designs can be included in the mapping [45]. This review revealed a paucity of methodologically strong studies, a lack of studies with social media as their main focus of interest, and further the studies were limited to specific regions of the world. Limitations in the included studies were the lack of theoretical frameworks and clear definitions of main concepts, and limited descriptions of data collection instruments, e.g. questions asked. Several of the studies suffer from deficiencies of various kinds, which makes it difficult to compare the results across the studies. It can explain the findings’ variety in the thematic analysis. Further, the current review cannot describe how and whether the nurses assess the gained information’s reliability, trustworthiness and usefulness. Furthermore, in several of the reviewed studies, it is difficult to separate nurses from healthcare professionals in general, which causes a risk that the focus on nurses specifically in the current review is blurred by other healthcare professions’ use of social media. In a scoping review, an optional element is a consulting exercise to inform and validate the findings, e.g. the consultants may provide additional references [37], which the current review did not comply with. A strength in the current study consists of the authors’ collaboration throughout the review, with a representation of senior researchers with different expertise areas.

## Conclusion

The current study provided some knowledge about nurses’ use of social media, although this did not come across as the main focus of the reviewed articles. The results showed that several healthcare professionals, including nurses, in the included studies used social media as channels to gain information about COVID-19 in relation to the pandemic. However, only few studies explored the healthcare professionals’ assessment of reliability and quality of this information and how/if it helped nurses gain adequate knowledge about COVID-19, in contrast to studies on physicians’ and pharmacists’ use of social media where quality of information was a more explicitly expressed concern. Social media was also used by nurses to share inconsistencies in COVID-19 advice and in that way sharpened attention to possible issues in the management of COVID-19. Moreover, nurses used social media to support each other by highlighting the need for training and changes in delivery of care and redeployment. Social media functioned as profession-promoting channels for nurses by on one hand sharing heroic self-representations and acknowledgment of frontline persons in the pandemic. In that way, social media was used to express solidarity between colleagues in healthcare. On the other hand, social media was used to display critical working conditions, and challenged or opposed current healthcare management. Finally, the study showed that nurses used social media to raise awareness and educate people to perform the ‘right ‘COVID-19’ behaviours in society. The reviewed studies provide snapshots from various regions in the world that, to various degrees, were struck by the COVID-19 pandemic. The studies may be of great value in the specific local context for policy, practice and pandemic preparedness in the healthcare sector. As most of the reviewed studies did not have social media as their main focus of interest even though it was mentioned in the abstract, they could hardly be blamed for not making more out of it. This study calls for further multi-methodological and in depth qualitative research with a specific focus on the role and uses of social media among nurses and other health professionals during the COVID-19 pandemic. The quantitative surveys dominating the current review provide us with some pieces of information. Yet there is a huge research gap concerning contextualised, rich, and nuanced descriptions of how healthcare professionals use social media, make sense of the COVID-19 information they come across on social media platforms, how they engage with the content, develop professional standards, construct a professional identity and use social media for contestation and development of clinical practices. In the quest for such knowledge, existent social media research and theories should be made better use of, guiding the studies and providing analytical perspectives for fruitful analysis and a more critical understanding of social media in a health and nursing context. On a final note, we conclude that there is also a need for more studies from other regions of the world, e.g. Europe and the Nordic countries, characterised by a high degree of digitalisation of healthcare and healthcare organisations.

## Supporting information

S1 Checklist Preferred Reporting Items for Systematic reviews and Meta-Analyses extension for Scoping Reviews (PRISMA-ScR) checklist section. (PDF)
